# Vav1 Selectively Down-Regulates Akt2 through miR-29b in Certain Breast Tumors with Triple Negative Phenotype

**DOI:** 10.3390/jpm12060993

**Published:** 2022-06-17

**Authors:** Silvia Grassilli, Federica Brugnoli, Stefano Cairo, Nicoletta Bianchi, Jean-Gabriel Judde, Valeria Bertagnolo

**Affiliations:** 1 Department of Translational Medicine, University of Ferrara, 44121 Ferrara, Italy; silvia.grassilli@unife.it (S.G.); bgf@unife.it (F.B.); nicoletta.bianchi@unife.it (N.B.); 2 LTTA Centre, University of Ferrara, 44121 Ferrara, Italy; 3 Xentech, 91000 Evry, France; stefano.cairo@xentech.eu (S.C.); jgjudde@xentech.eu (J.-G.J.); 4 Istituto di Ricerca Pediatrica, 35127 Padova, Italy

**Keywords:** triple negative breast cancer (TNBC), miR-29b, Vav1, Akt2, targeted therapy

## Abstract

Triple negative breast cancer (TNBC) represents the most aggressive breast tumor, showing a high intrinsic variability in terms of both histopathological features and response to therapies. Blocking the Akt signaling pathway is a well-studied approach in the treatment of aggressive breast tumors. The high homology among the Akt isoforms and their distinct, and possibly opposite, oncogenic functions made it difficult to develop effective drugs. Here we investigated the role of Vav1 as a potential down-regulator of individual Akt isozymes. We revealed that the over-expression of Vav1 in triple negative MDA-MB-231 cells reduced only the Akt2 isoform, acting at the post-transcriptional level through the up-modulation of miR-29b. The Vav1/miR-29b dependent decrease in Akt2 was correlated with a reduced lung colonization of circulating MDA-MB-231 cells. In cell lines established from PDX, the Vav1 induced down-modulation of Akt2 is strongly dependent on miR-29b and occurs only in some TNBC tumors. These findings may contribute to better classify breast tumors having the triple negative phenotype, and suggest that the activation of the Vav1/miR-29b axis, precisely regulating the amount of an Akt isozyme crucial for tumor dissemination, could have great potential for driving more accurate therapies to TNBCs, often not eligible or resistant to treatments.

## 1. Introduction

Triple negative breast cancer (TNBC) accounts for 10–20% of all mammary tumors and represents the most aggressive subtype of breast cancer, characterized by high cell proliferation and metastasis and poor prognosis [1]. In recent years, TNBC was recognized as a heterogeneous group of malignancies, and various molecular and morphological stratification systems have been proposed to reflect its intrinsic variability. At present, molecular and morphological classifications identify TNBCs with distinct biological behaviors resulting in different histopathology, tumor grade and disease progression rate [1,2,3,4,5,6]. At variance with other breast tumors, TNBC lacks specific target molecules, and chemotherapy still represents the only effective therapeutic strategy. Unfortunately, the drug resistance occurring after the long-term use of chemotherapeutic agents combined with the heterogeneous response to treatments contribute to make this approach ultimately unsuccessful [1,6].

Hyperactivation of Akt signaling is a central event in human cancer and almost 30% of TNBCs have alterations in the PI3K/Akt/mTOR pathway [7,8]. The Akt family includes three members (Akt1, Akt2 and Akt3) that play distinct roles in breast cancer malignancy, being Akt1 correlated with proliferation of tumor cells and the other two isozymes variously involved in invasion/metastasis [9,10,11]. In tumors with a triple negative phenotype, Akt2 has a crucial role in metastasis by regulating the epithelial-to-mesenchymal-transition (EMT) and by enabling extravasation through the vessel wall [12]. Furthermore, the silencing of Akt2 in triple negative breast tumor cells reduces the population and the metastatic potential of cancer stem cells (CSCs) [13,14,15].

Among the signaling molecules involved in the regulation of Akt isozymes in breast tumors, a crucial role was recently demonstrated for Vav1, a multidomain protein physiologically expressed in hematopoietic cells, in which it regulates cytoskeleton reorganization, gene transcription and miRNAs expression [16,17,18]. Vav1 is ectopically expressed in breast tumor cells, including TNBC derived cells, in which it promotes the down-modulation of activated Akt1, resulting in reduced tumor growth in vivo [19,20]. On the other hand, the over-expression of Vav1 in TNBC cells also reduces their invasive potential in vitro and their metastatic efficiency in vivo, and its silencing up-modulates the expression of Akt2 [20].

Accumulating evidence revealed that, in breast cancer as in other solid tumors, Akt2 levels may be modulated by several miRNAs [21], including miR-615, which was reported to reduce the expression of Akt2 in TNBC cells [22]. In the same breast tumor, it was recently demonstrated that miR-29b down-regulates cells growth in 3D cultures by abrogating the expression of different oncogenes including Akt2 [23].

As we revealed that in TNBC cells with a mesenchymal phenotype, Vav1 positively regulates the expression of miR-29b by acting at transcriptional level [24], the present work was aimed to establish whether Vav1 down-modulates the expression of Akt2 through miR-29b, to hopefully identify a Vav1/miR-29b/Akt2 axis to be targeted for a more efficient and subtype-specific therapeutic strategy of tumors with a triple negative phenotype.

## 2. Materials and Methods

All reagents were obtained from Merck KGaA unless otherwise indicated.

- Cell lines

MDA-MB-231 cells were purchased from the American Type Culture Collection (Rockville, MD, USA) and were maintained in Dulbecco’s modified Eagle’s medium (DMEM, Gibco Laboratories, Grand Island, NY, USA) supplemented with 10% fetal bovine serum (FBS, Gibco Laboratories, Grand Island, NY, USA) and 1% penicillin-streptomycin (Gibco Laboratories, Grand Island, NY, USA). Established cell lines from patient-derived xenografts (PDXs) of breast tumors with a triple-negative phenotype (HBCx-2, HBCx-9, HBCx-17, HBCx-39 and T174) were provided from Xentech (Evry, France) and cultured as previously described [24]. All cell lines were cultured at 37 °C in a humidified atmosphere and evaluated monthly for mycoplasma and other contaminations.

- Modulation of Vav1 and miR-29b expression

The modulation of Vav1 was performed following previously reported procedures [24]. For down-modulation of Vav1, a mix of specific siRNAs was used (Santa Cruz Biotechnology, Santa Cruz, CA, USA) and the Vav1 over-expression was obtained through transient transfection with a plasmid expressing the human full-length Vav1. Non-silencing siRNAs (Santa Cruz Biotechnology, Santa Cruz, CA, USA) and an empty vector were used for control transfection experiments. Transfected cells were incubated at 37 °C in a 5% CO_2_ atmosphere for 48 h and then subjected to immunochemical and RT-qPCR analysis.

To obtain MDA-MB-231 cells stably expressing Vav1, cells transfected with a vector concomitantly expressing the human Vav1 and a neomycin resistance gene (neo), enabling the cells to grow in medium containing Geneticin, were cultured for 3 weeks in the presence of 1 mg/mL G418, as previously reported [19]. The resistant cells, over-expressing Vav1, were maintained in culture with a medium containing 0.1 mg/mL G418, and then used for in vivo experiments.

For specific modulation of miR-29b-3p, transient transfections were performed with a synthetic inhibitor or mimic (miRVana miRNA, Thermo Fisher Scientific, Waltham, MA, USA), as previously reported [25].

- Animal models

Xenografts from MDA-MB-231 cells were obtained injecting subcutaneously into BALB/c nude mice (female, age six weeks, *n* = 10 per group, Charles River Laboratories Italia, Lecco, Italy) cells stably over-expressing Vav1 or an empty vector, as previously described [20]. Throughout the experiments, mice were maintained with free access to pellet food and water. After 8 weeks, the mice were euthanized with inhalation of CO_2_ and tumor xenografts were removed and fixed with 4% paraformaldehyde. By using a Leica microtome (Leica Biosystems, Wetzlar, Germany), 5 µm tissue sections from paraffin-embedded xenografts were obtained, that were subjected to immunohistochemical analysis of Akt2. 10 µm sections were laser micro-dissected to evaluate miR-29b expression.

To produce experimental lung metastasis, 6-week-old female BALB/c nude mice (*n* = 7 per group) were injected intravenously with MDA-MB-231 cells over-expressing human Vav1 or the empty vector (2 × 10^6^ in 0.1 mL PBS) via tail veins [19]. After 12 weeks, the mice were euthanized, and the lungs were removed and fixed with 4% paraformaldehyde. Paraffin embedded sections were stained with hematoxylin-eosin (HE) and the presence of discrete tumors was evaluated. Paraffin embedded sections of lung from mice receiving cells transfected with the empty vector were subjected to immunohistochemical analysis of Vav1 and Akt2.

- Reverse transcription quantitative PCR (RT-qPCR)

High-quality RNA, including small RNAs, was extracted from all cell lines using miRNeasy Micro Kit (Qiagen SpA, Milan, Italy), according to the manufacturer’s instructions. The expression of Akt1 (ID Hs00178289_m1), Akt2 (ID Hs00609846_m1) and Akt3 (ID Hs00987343_m1) was evaluated by quantitative Real-Time PCR. mRNA levels were calculated by the 2^−ΔCt^ method and normalized to RPL13A (ID Hs03043885), as previously described [26].

MiR-29b-3p (ID 000413, Thermo Fisher Scientific, Inc., Waltham, MA, USA) and miR-615-3p (ID 001960, Thermo Fisher Scientific, Inc., Waltham, MA, USA) levels were evaluated by RT-qPCR using TaqMan Assays, and normalized to U6 snRNA (ID 001973, Thermo Fisher Scientific, Inc., Waltham, MA, USA), as previously described [24]. Fold changes were determined using the 2^−ΔΔCt^ method.

- Laser-capture micro-dissection and analysis of miR-29b expression

Sections of breast tumor xenografts were laser micro-dissected (LMD) by using the LEICA LMD 6500 laser microdissector (Leica Microsystems, Wetzlar, Germany), as previously reported [25]. For each sample, areas of 2–20 × 10^6^ μm^2^ were selected and micro-dissected samples were subjected to microRNAs extraction by using the miRNeasy FFPE kit (Qiagen, Milan, Italy). RNA was reverse-transcribed for miR-29b-3p using the TaqMan MicroRNA RT kit (ThermoFisher Scientific). The miRNA was quantified by Real-Time qPCR using TaqMan MicroRNA assays (ID 000413, Thermo Fisher Scientific, Inc., Waltham, MA, USA) and expression levels were normalized to U6 snRNA (ID 001973, Thermo Fisher Scientific, Inc., Waltham, MA, USA). Fold change was determined using the 2^−ΔΔCt^ method.

- Immunochemical analysis

Total cell lysates were separated on 7.5% polyacrylamide denaturizing gels and blotted on nitrocellulose membranes (GE Healthcare Life Science, Little Chalfont, UK), that were reacted with antibodies directed against Vav1 (sc-8039), Akt1 (sc-1618), Akt2 (sc-5270) and Akt3 (sc-134254) (Santa Cruz Biotechnology, Santa Cruz, CA, USA), against p-Akt2 (Ser474, ≠8599) Cell Signalling Technology, Danvers, MA, USA), and against β-Tubulin (T4026), following previously reported procedures [20]. The immunocomplexes were detected by using the ECL system (Perkin-Elmer, Boston, MA, USA), and the chemiluminescence derived bands were acquired with an ImageQuantTM LAS 4000 imager (GE Healthcare Life Science, Little Chalfont, UK) and quantified by means of Image Quant TL software (GE Healthcare Life Science, Little Chalfont, UK), as previously reported [24].

- Immunohistochemical analysis

Immunohistochemical staining of Akt2 and/or Vav1 in tumor xenografts and lung sections was performed with anti-Akt2 and anti-Vav1 antibody (Santa Cruz Biotechnology, Santa Cruz, CA, USA), respectively, using the UltraVision LPValue Detection System-HRP Polymer (Ready-To-Use) (Thermo Fisher Scientific, Waltham, MA, USA), as previously reported [27]. Slides were subjected to a block of endogenous peroxidases, and the Ultra V Block reagent (Thermo Fisher Scientific, Waltham, MA, USA) was used to reduce background. The samples were then incubated over night at 4 °C with anti-Akt2 or an anti-Vav1 antibody in a large volume of UltrAbDiluent (Thermo Fisher Scientific, Waltham, MA, USA). Staining was detected by the addition of a substrate/chromogen mix (DAB Quanto, Thermo Fisher Scientific, Waltham, MA, USA) and the tissue sections were counterstained with Mayer’s hemalum (Bio-Optica, Milan, I). Negative controls were obtained by omitting the primary antibody. Stained tissue samples were analyzed by a Nikon Eclipse 90i (Nikon, Melville, NY, USA), and cell images were acquired with the NIS-ElementsF 2.30 software for a DS-2Mv digital camera (Nikon, Melville, NY, USA).

For each tumor xenograft or lung section, at least three different regions containing at minimum 100 cells were analyzed, and the staining intensity was evaluated with the ImageScope software (Aperio, Vista, CA, USA) and quantified as Intensity of Positive pixel/Area (IP/µm^2^), as previously reported [27].

- Real-time cell invasion assays

Cell invasion was evaluated by means of the xCELLigence Real-Time Cell Analyzer System (RTCA System, Roche Applied Science, Mannheim, Germany), developed to monitor cell events in real time, as previously reported [28]. 40.000 cells/well were seeded onto the top chambers of CIM-16 plates (Roche Applied Science, Mannheim, Germany) covered with Matrigel (BD Biosciences, San Josè, CA, USA) diluted 1:20. The bottom chambers were filled with medium containing 10% serum and the signal detection was automated every 15 min for a total of 24 h. Impedance values were expressed as the dimensionless parameter Cell Index (CI).

- Statistical analysis

The results were expressed as means ± standard deviations of three independent experiments. Statistical analysis was performed by using the 2-tailed Student’s t test for unpaired data calculated with the GraphPad Prism 6.0 statistical package (GraphPad Software, San Diego, CA, USA). *p* values < 0.05 were considered statistically significant.

## 3. Results

### 3.1. Vav1 Regulates Akt2 through miR-29b in MDA-MB-231 Cells

**Figure 1:**
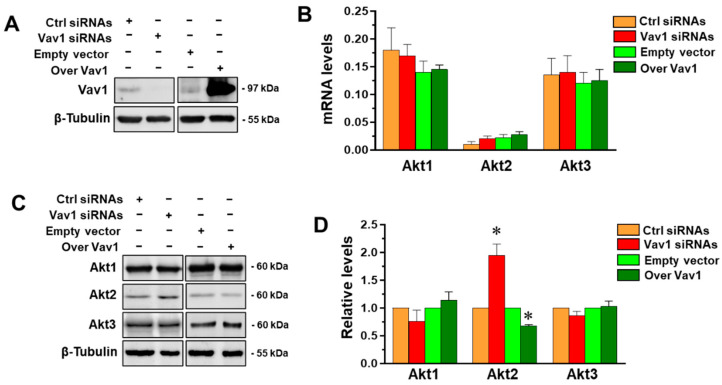
Correlation between Vav1 and Akt isoforms in MDA-MB-231 cells. (**A**) Representative immunochemical analysis of lysates from MDA-MB-231 cells transfected with siRNAs specific for Vav1 (Vav1 siRNAs) or with a construct expressing Vav1 (Over Vav1). Scramble siRNAs (Ctrl siRNAs) and an empty vector were used as controls. β-Tubulin was blotted as an internal control of loaded proteins. (**B**) qRT-PCR analysis of mRNA (2^−ΔCt^ method) and (**C**) representative Western blot of Akt1, Akt2 and Akt3 in MDA-MB-231 cells cultured under the above reported experimental conditions. (**D**) Levels of Akt isoforms as deduced from the chemiluminescence signal normalized with β-Tubulin. Asterisks indicate statistically significant differences with respect to corresponding controls taken as 1. All the data are the means of 3 separate experiments ± SD. * *p* < 0.05.

The role of Vav1 in modulating the expression of the three Akt isoforms was firstly investigated, in terms of both mRNA and protein levels, in the triple negative MDA-MB-231 cells in which Vav1 was down-modulated or over-expressed (Figure 1A). As shown in Figure 1B, MDA-MB-231 cells express the members of the Akt family at various levels, Akt1 being the most abundant and Akt2 the least expressed.

Concerning the effects of Vav1 modulation, none of the mRNAs for the three Akt isozymes were significantly modified when Vav1 was over-expressed or down-modulated (Figure 1B), excluding its role in regulating Akt at the transcriptional level. When the protein amounts of the three isoforms were investigated by Western blot analysis, we revealed that only the expression of Akt2 was affected by the forced modulation of Vav1. In particular, the silencing of Vav1 increased while its over-expression induced a significant decrease in Akt2 expression (Figure 1C,D), suggesting that Vav1 may modulate the level of this Akt isoform at post-transcriptional level.

**Figure 2:**
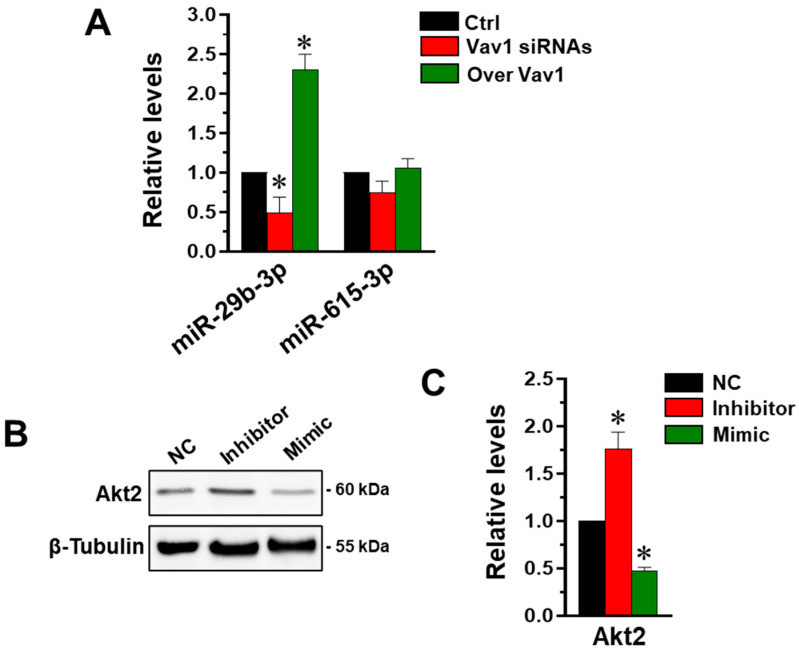
Correlation between Vav1, miR-29b and Akt2 in MDA-MB-231 cells. (**A**) qRT-PCR analysis of miR-29b-3p and miR-615-3p in MDA-MB-231 cells in which Vav1 was down-modulated or over-expressed. The values were determined using the 2 ^−ΔΔCT^ method and the asterisks indicate statistically significant differences compared to respective controls (Ctrl) taken as 1. (**B**) Representative immunoblot analysis of Akt2 in lysates from MDA-MB-231 cells transfected with miR-29b-3p inhibitor or mimic. In (**C**), amounts of Akt2 as deduced from the chemiluminescence signals normalized with β-Tubulin. The asterisks indicate significant differences compared to transfection with scramble oligonucleotides (Negative control, NC), taken as 1. All the data represent the mean of three separate experiments ± SD. * *p*  <  0.05.

As it is known that, in breast cancer, Akt2 is regulated by various miRNAs, including miR-615 [22] and miR-29b [23], we explored their possible involvement in the mechanism by which Vav1 affects this Akt isoform. We firstly evaluated the role of Vav1 in modulating the level of the two miRNAs by performing a PCR analysis on MDA-MB-231 cells in which the protein was forcedly over-expressed or silenced. We revealed that miR-615 was not significantly affected by levels of Vav1 (Figure 2A), and therefore probably not involved in the Vav1-dependent modulation of Akt2. The analysis of miR-29b confirmed its relationship with Vav1 levels [24], as deduced by the decrease in the miRNA expression in Vav1 silenced cells, and by its up-modulation in cells in which Vav1 was over-expressed (Figure 2A).

Once confirmed that, in addition to Akt2, Vav1 regulates miR-29b in MDA-MB-231 cells, we evaluated the role of this miRNA in modulating the Akt isoform in this cell model. The MDA-MB-231 cell line was then treated with synthetic mimic and inhibitors specific for miR-29b, and the levels of Akt2 were evaluated by Western blot. As shown in Figure 2B,C, the silencing of miR-29b resulted in an increase in Akt2 levels and its up-regulation down-modulated the protein amount.

### 3.2. Vav1 Sustains the miR-29b/Akt2 Axis in MDA-MB-231 Cells In Vivo

**Figure 3:**
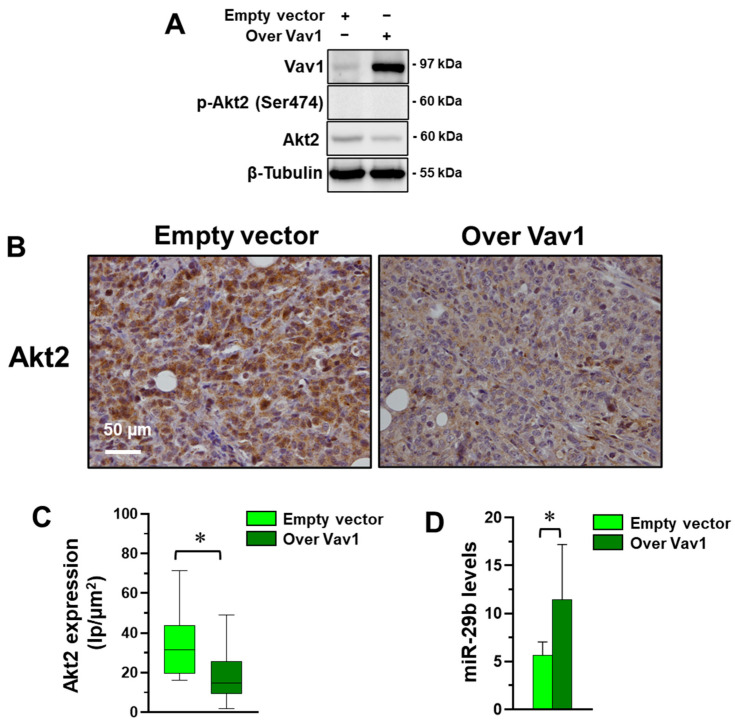
Correlation between Vav1, miR-29b and Akt2 in MDA-MB-231-derived xenografts. (**A**) Representative immunochemical analysis of MDA-MB-231 cells stably expressing an empty vector or Vav1. (**B**) Representative immunohistochemical analysis with the anti-Akt2 antibody of xenografts from the above-described MDA-MB-231 cells. (**C**) Analysis of Akt2 staining in xenografts that reports the Intensity of Positive pixels/Area (Ip/µm^2^). (**D**) qRT-PCR analysis (2^−ΔCt^) of miR-29b-3p in laser micro-dissected xenografts sections. * *p* < 0.05 between bars.

Once we assessed the existence of a correlation between Vav1, miR-29b and Akt2 in MDA-MB-231 cells in vitro, we tried to establish if this relationship also exists in vivo. Xenografts were then obtained by injecting subcutaneously into nude mice MDA-MB-231 cells stably over-expressing human Vav1, showing lower levels of Akt2, that resulted non-phosphorylated in this cell model (Figure 3A). Tumors formed in the subcutaneous skin layer were analyzed for Akt2 levels by immunohistochemical analysis, revealing the significantly lower staining of Akt2 in xenografts from mice injected with MDA-MB-231 stably over-expressing Vav1 compared with tumors from mice injected with cells transfected with an empty vector (Figure 3B,C). The same areas of all samples analyzed for the Akt2 content were subjected to laser micro-dissection and to qRT-PCR analysis of miR-29b, revealing higher levels of the miRNA in tumors derived from MDA-MB-231 cells over-expressing Vav1 (Figure 3D).

**Figure 4:**
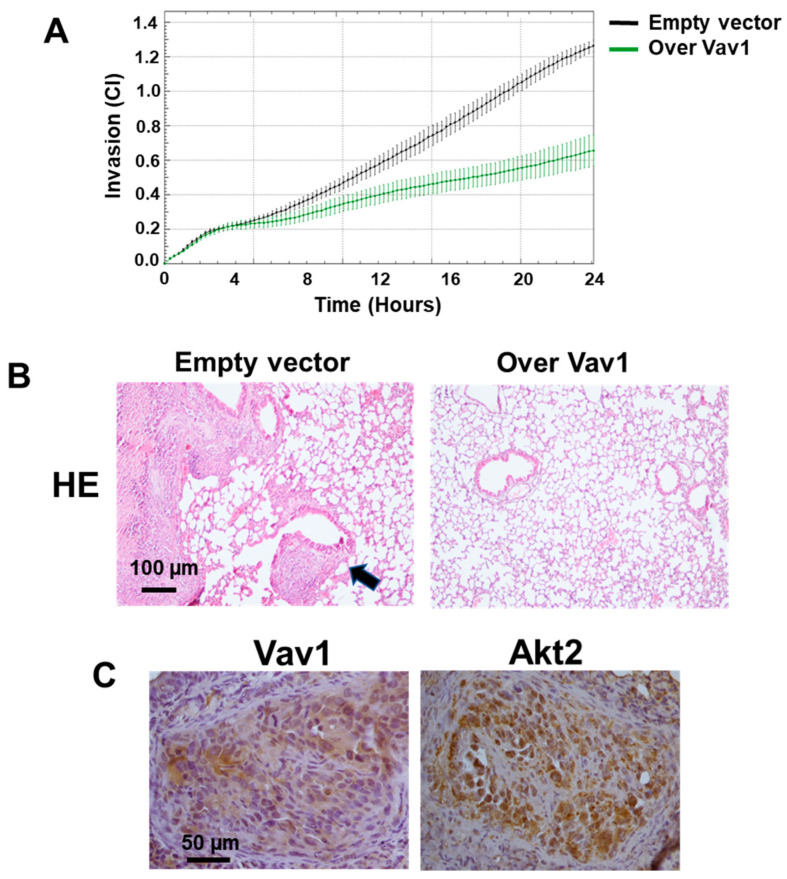
Effects of Vav1 on lung colonization of MDA-MB-231 cells. (**A**) Dynamic monitoring of invasiveness of MDA-MB-231 cells stably expressing an empty vector or Vav1. Cell Index (CI) values are reported, and error bars indicate SD. (**B**) Representative HE images of lung sections from mice following intravenous injection of MDA-MB-231 cells transfected with an empty vector or stably expressing Vav1. The arrow indicates a lung secondary tumor, whose immunohistochemical analysis of Vav1 and Akt2 was shown in (**C**).

As the MDA-MB-231 cells in which Vav1 was forcedly expressed, having higher miR-29b (Figure 2A) and lower Akt2 (Figure 1B,C) showed a reduced invasive potential (Figure 4A), and the Vav1/miR-29b/Akt2 axis was further correlated with the ability of MDA-MB-231 cells to form secondary tumors in vivo. After 12 weeks from tail vein injection, no detectable macro-metastases were observed on the surface of lungs from animals (data not shown). Nevertheless, the analysis of HE-stained sections revealed tumor cell aggregates in lungs from mice receiving empty vector transfected cells, two of which developed lung macro-metastases (Figure 4B). On the contrary, none of the mice receiving MDA-MB-231 cells over-expressing Vav1 developed visible lung cell aggregates (Figure 4B), suggesting that the Vav1-induced miR-29b, in turn involved in down-modulation of Akt2, counteracted the metastatic efficiency of MDA-MB-231 cells. The immunohistochemical analysis of lung tumor masses confirmed the presence of Akt2 and Vav1 of human origin in these secondary tumors (Figure 4B).

### 3.3. Vav1 Regulates the miR-29b/Akt2 Axis in Cells from Patient-Derived Xenografts

**Figure 5:**
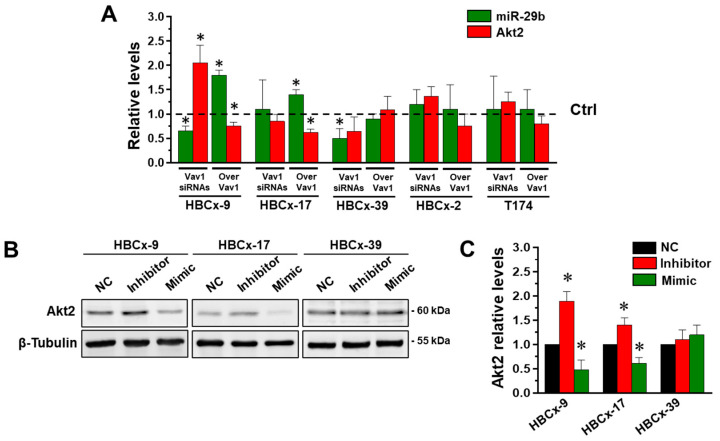
Correlation between miR-29b and Akt2 in cell lines from triple negative PDXs. (**A**) Amounts of miR-29b and Akt2 in PDX-derived cell lines in which Vav1 was silenced or over-expressed. The values are relative to those of cells transfected with control siRNAs or empty vectors (Ctrl), respectively, taken as 1. * *p* <0.05. (**B**) Representative Western blot analysis of PDX-derived cell lines in which miR-29b-3p was down- (Inhibitor) or up-modulated (Mimic). In (**C**), amounts of Akt2 as deduced from the chemiluminescence signal normalized with β-Tubulin. * *p*  <  0.05 compared to transfection with scramble oligonucleotides (NC). All the data are the means of 3 separate experiments ± SD.

In cell lines established from patient-derived xenografts (PDX) from TNBC tumors belonging to different molecular subtypes, we have previously demonstrated that Vav1 is able to up-modulate miR-29b only in cells expressing the transcription factor CEBPα [24]. To establish if the relationship between Vav1 and Akt2 in TNBC is strictly dependent on mir-29b and/or if it can correlate with specific molecular subtype/s, PDX-derived cell lines, belonging to the refined TNBCtype-4 subtypes identified by Lehmann et al. [2], were subjected to modulation of Vav1 and evaluated for their expression of miR-29b and Akt2. As expected [24], we revealed that the silencing of Vav1 reduced miR-29b in HBCx-9 (BL1), and HBCx-39 (BL1) cells and that the over-expression of the protein induced the miRNA in the HBCx-9 and HBCx-17 (M) cell lines (Figure 5A), without significant effects of the Vav1 modulation in the HBCx-2 (LAR) and T174 (M) cell lines. The analysis of Akt2 expression revealed a significant reduction in the protein only in the HBCx-9 and HBCx-17 cells in which Vav1 was over-expressed, in accordance with the increase in miR-29b levels (Figure 5A). On the other hand, the expression of Akt2 significantly increased only in HBCx-9, in which the silencing of Vav1 diminished miR-29b expression (Figure 5A), demonstrating the existence of a Vav1/miR-29b/Akt2 axis in some breast tumor cells with a triple negative phenotype, apparently unrelated to a specific molecular subtype.

To verify the existence of a relationship between miR-29b and Akt2 in cells in which Vav1 regulated Akt2, HBCx-9, HBCx-17 and HBCx-39 cells were subjected to modulation of miR-29b by using a synthetic mimic and inhibitors, and evaluatd for Akt2 expression. As shown in Figure 5B,C, in both the HBCx-9 and HBCx-17 but not in the HBCx-39 cell lines, the inhibition of miR-29b induced the expression of Akt2 and its up-modulation reduced the protein level, clearly indicating that the Vav1 dependent regulation of Akt2 in triple negative breast tumor cells depends on its ability to modulate miR-29b levels.

## 4. Discussion

The data reported here demonstrated a peculiar role of the multidomain protein Vav1 in breast tumor cells with a triple negative phenotype, consisting in down-modulation of the oncogenic protein Akt2 through the up-modulation of the tumor suppressor miR-29b.

The ectopic expression of Vav1 in breast tumor cells with different phenotypes was demonstrated by different groups [19,29,30,31], but the only nuclear localization of this protein was positively correlated with prognosis [19], suggestive of an antitumor role of Vav1 dependent on its activity inside the nuclear compartment. The nuclear role of Vav1 was mainly investigated in leukemic cells, in which the protein was found to cooperate with transcription factors to modulate the expression of differentiation related genes and miRNAs [17,18,32].

In breast tumor cells, the nuclear content of Vav1 was correlated with a phenotype-specific expression of genes involved in tumor progression, including precise Akt isozymes [19]. Silencing of Vav1 resulted in the increased expression in Akt1 in ER+ cells and in the up-modulation of Akt2 in ER- breast tumor cells, including those with a triple negative phenotype [20], still representing the most aggressive breast cancer [1]. TNBC shows a higher proliferation rate and higher incidence of metastases than the other breast cancer subtypes, and the absence of expression of ER, PR, and/or over-expression of HER2, leave chemotherapy the only, often ineffective therapeutic alternative for this neoplasia [1,4].

Akt is hyperactivated in most breast cancers and, in recent years, several inhibitors that block the phosphorylation of the enzyme have been identified, despite the high homology among the Akt isozymes which made it difficult to develop isoform-specific drugs [33]. In breast tumors as in other cancers, growing evidence shows distinct and perhaps opposite oncogenic functions of Akt isozymes, the expression of which may also vary in primary and secondary sites of tumors [11,15]. Based on the need of precise isoform-specific inhibitors, especially in the treatment of the most aggressive breast cancers, the role of Vav1 as a potential down-regulator of individual Akt isozymes was investigated in the MDA-MB-231 cell line, highly invasive and with a triple negative phenotype. We confirmed that, of the three Akt isozymes, only levels of Akt2 were affected by the forced modulation of Vav1 in TNBC cells [20]. On the other hand, the lack of effects on the mRNA for this Akt isozyme reasonably ruled out the role of Vav1 in regulating Akt2 expression at the transcriptional level, suggesting its involvement in the post-transcriptional modulation of the protein amount.

Accumulating evidence showed that miRNAs play a crucial role in the pathogenesis of tumors, and numerous miRNAs responsible for modulation of Akt2 resulted in important diagnostic, prognostic, and therapeutic markers in various cancers [21]. Our further investigations have been therefore oriented to establish whether, in breast tumor as in leukemic cells [18], Vav1 can modulate the expression of miRNA/s, in turn responsible for targeting Akt2. Our experiments excluded the involvement of miR-615, targeting Akt2 in MDA-MB-231 and pancreatic cancer cells [21,22], as it was not modified by Vav1 levels in this cell model. To explore the involvement of other miRNA/s with a known role in targeting Akt2, we focused on miR-29b, whose gene transcription is positively regulated by Vav1 in acute myeloid leukemia [18] and in MDA-MB-231 cells [24] and that is known to target Akt2 in glioblastoma, gastric and ovarian cancers [21,34]. We demonstrated here the ability of the Vav1-induced miR-29b to down-regulate Akt2 in MDA-MB-231 cells both in vitro and in tumor xenografts derived from MDA-MB-231 cells stably over-expressing Vav1, ascertaining the existence of a Vav1/miR-29b/Akt2 axis in these TNBC cells.

Despite that a consensus regarding the isoform-specific functions of Akt was not reached even within a specific subtype of breast cancer, Akt2 was associated with tumor progression by increasing cell migration, invasion, and metastasis [9,10]. It was further demonstrated that Akt2 prevents apoptosis and promotes lung colonization of circulating TNBC cells [12]. With the aim to assess if the Vav1 dependent miR-29b/Akt2 relationship is involved in lung colonization of TNBC cells, MDA-MB-231 stably over-expressing Vav1, in which high miR-29b and low Akt2 levels correlated with reduced invasion capability in vitro, were injected into mice tail veins and evaluated for their ability to form lung tumors. The evidence that none of the mice receiving cells over-expressing Vav1 developed lung aggregates of breast tumor cells suggests a protective role of the Vav1-induced miR-29b against the facilitation of extravasation of tumor cells into lung parenchyma, which was proposed to correlate with Akt2 [12]. Moreover, our finding that Akt2 expression but not phosphorylation is down-modulated by Vav1 in MDA-MB-231 cells [20], supports the evidence that Akt2 facilitates TNBC lung colonization independent of its activity [12]. This phenomenon, attributed to the eventual compensatory phosphorylation of the other Akt isoforms present within the cells or to a non-kinase function of Akt2, confirmed the need to identify molecules capable of specifically inhibiting the individual Akt isozymes. In this context, the activation of the Vav1/miR-29b axis, ended to precisely regulate the amount of Akt2, may constitute a specific approach for TNBCs, which are often not eligible or resistant to current therapeutic regimens.

TNBC is a highly heterogeneous neoplasia, and in the last ten years, breast tumors with a triple negative phenotype were classified based on their histological, genetic, and epigenetic characteristics [2,3,35]. In a recent study performed on PDX-established cell lines from triple negative breast tumors we demonstrated that Vav1 promotes the expression of miR-29b exclusively in cells showing relatively high levels of CEBPα [24], the main transcription factor for the miR-29b1/a cluster [36], whose activity is strongly dependent on Vav1 levels [24]. Here we demonstrated that, in PDX-derived cell lines belonging to the most frequent TNBC molecular subtypes identified by Lehmann [2], one of the most studied classifications as it recognized tumors that differ in histopathology, response to neoadjuvant chemotherapy [37,38,39] and disease progression, forcedly expressed Vav1 significantly down-modulates Akt2 only in cells in which it up-modulates miR-29b. This occurrence does not appear to correlate with specific Lehmann subtype/s, and contributes to further increase the intrinsic variability of breast tumors with a triple negative phenotype.

Despite the advent of new sequencing technologies and of advanced computing systems allowed to increase the number of data to be used for cataloging, TNBC subtypes identified with the different methods are often overlapped, and however they fail to precisely classify every single tumor [38,39]. This, on one hand, confirms that specific profiles can define the response to therapy and, ultimately, the prognosis of TNBC [2,4,5]. On the other hand, it highlights the requiring of an accurate characterization of each tumor to choose the more effective therapeutic strategy. Our data, clearly indicating the existence in some TNBC of a Vav1/miR-29b/Akt2 connection able to precisely down-modulate an Akt isozyme crucial for tumor dissemination, may contribute to better classify breast tumors and suggest that therapies aimed to activate the Vav1/miR-29b axis could have great potential for a more personalized therapeutic approach for breast tumors with a triple negative phenotype.

## Data Availability

Not applicable.
